# Identification and characterization of MDR virulent *Salmonella* spp isolated from smallholder poultry production environment in Edo and Delta States, Nigeria

**DOI:** 10.1371/journal.pone.0281329

**Published:** 2023-02-03

**Authors:** Isoken H. Igbinosa, Chukwunonso N. Amolo, Abeni Beshiru, Olajide Akinnibosun, Abraham G. Ogofure, Maged El-Ashker, Mayada Gwida, Anthony I. Okoh, Etinosa O. Igbinosa

**Affiliations:** 1 Department of Environmental Management & Toxicology, Faculty of Life Sciences, University of Benin, Benin City, Nigeria; 2 Applied Microbial Processes & Environmental Health Research Group, Faculty of Life Sciences, University of Benin, Benin City, Nigeria; 3 Stellenbosch Institute for Advanced Study (STIAS), Wallenberg Research Centre at Stellenbosch University, Stellenbosch, South Africa; 4 Department of Internal Medicine and Infectious Diseases, Faculty of Veterinary Medicine, Mansoura University, Mansoura, Egypt; 5 Department of Hygiene and Zoonoses, Faculty of Veterinary Medicine, Mansoura University, Mansoura, Egypt; 6 Department of Environmental Health Sciences, College of Health Sciences, University of Sharjah, Sharjah, United Arab Emirates; 7 SAMRC Microbial Water Quality Monitoring Centre, University of Fort Hare, Alice, Eastern Cape Province, South Africa; North Carolina State University, UNITED STATES

## Abstract

*Salmonella* is responsible for some foodborne disease cases worldwide. It is mainly transmitted to humans through foods of animal origin through the consumption of poultry products. The increased international trade and the ease of transboundary movement could propel outbreaks of local origin to translate into severe global threats. The present study aimed to characterize *Salmonella* serovars isolated from poultry farms in Edo and Delta States, Nigeria. A total of 150 samples (faecal, water and feed) were collected from ten poultry farms between January and August 2020 and analyzed for *Salmonella* characterization using standard bacteriological and molecular methods. *Salmonella* serovars identified include: *Salmonella* Enteritidis [*n* = 17 (39.5%)], *Salmonella* Typhimurium [*n* = 13 (30.2%)] and other *Salmonella* serovars [*n* = 13 (30.2%)]. All *Salmonella* serovars were cefotaxime and ampicillin resistant. The presence of the *invA* gene ranged from 9(69.2%) to 15(88.2%). The *spvC* gene ranged from 2(14.4%) to 10(58.8%). All *Salmonella* serovars had *sdiA* gene. The *Salmonella* isolates produced some extracellular virulence factors (such as protease, lipase, β-hemolytic activity, and gelatinase), while 13(30.2%) of the overall isolates formed strong biofilms. In conclusion, the detection of multiple antibiotic-resistant *Salmonella* serovars in faecal sources, which also exhibited virulence determinants, constituted a public health risk as these faecal samples have the potential as manure in the growing of crops. These pathogens can be transmitted to humans nearby and through poultry products, resulting in difficult-to-treat infections and economic loss.

## Introduction

*Salmonella* is a Gram-negative bacterial genus globally documented as a significant pathogen of zoonotic concern for both animals and humans. Above 2500 *Salmonella* serovars are globally distributed [1]. *Salmonella* is responsible for most foodborne disease cases worldwide. It is mainly transmitted to humans through foods of animal origin, such as eggs, milk, and meat [2]. The detection of *Salmonella* spp., in foodstuff obtained from poultry products increased in the 1980s, with *Salmonella* Enteritidis responsible for numerous foodborne disease outbreaks in England due to the consumption of food containing poultry ingredients [2]. There have been several cases of foodborne disease outbreaks in humans due to the consumption of poultry products in the 1990s [3].

*Salmonella* in poultry is a problem in developing countries [4] and developed countries [5–7]. In Nigeria, salmonellosis has been reported in asymptomatic carriers, poultry farms, food consumers and children [8, 9]. In the European Union (EU), salmonellosis was considered the most reported infection of zoonotic concern in 2009, with about 108,614 confirmed human cases with a death rate of 0.08%, corresponding to about 90 human mortalities [10]. The increase in global trade and the ease of transboundary movement could help the dissemination of contaminants and pathogenic agents in foodstuffs and vulnerable humans. Currently, the world is interdependent and interrelated. Thus, outbreaks of local origin could translate to severe threats globally [11]. Commercialization, globalization, and spread enhance and enable contaminated foods to simultaneously affect people in different countries. Hence, surveillance systems and food safety measures need to be improved to identify foods involved in disease outbreaks [2].

Antimicrobial resistance (AMR) has become a severe menace [12]. Multidrug resistance (MDR) has been detected in several *Salmonella* serovars [6], which have been linked to the increase in hospitalization, deaths and the cost of treatment [13]. Recent studies have shown increased antibiotic resistance to *Salmonella* serovars recovered from humans and animals [14]. Growth promoters in animal feeds and inappropriate use of antibiotics for therapeutics and prophylactics resulted in resistance among *Salmonella* strains [15]. High antimicrobial usage, including the highest priority critically important antimicrobials, has been observed at poultry farms in Nigeria [16]. The high levels of resistance to tetracycline, sulfonamides, ciprofloxacin and gentamicin in *Salmonella* serovars correlated to the high farm-level usage of these antimicrobials there was a strong correlation between the number of antimicrobials used and resistance of isolates to the same antimicrobials [17]. Indiscriminate use of antimicrobials by farmers and the potential risk of AMR within the smallholder poultry production systems in Nigeria have been reported [18–20]. Growth promoters are still allowed in Nigeria even though they have been banned in the EU since 2006 and pose a high risk for emerging antibiotic resistance [19, 20].

Several antimicrobial resistance genes (ARGs) are located in the genomic island of *Salmonella* serovars, and mobile genetic elements (MGEs) such as integrons and plasmids aid the dissemination of resistance elements between different strains and aid the selection of resistant mutants within the population [21]. When more pathogenic bacterial strains become resistant to antimicrobials, the drugs become less effective and suitable for treatment options. *Salmonella* easily forms biofilm on contact surfaces [22]. Once the biofilm is created, it protects the embedded bacteria from external physical and chemical treatment [23], which can ultimately aid its transmission to humans in close contact. Biofilm formation is essential for spreading *Salmonella* serovars due to their resistance to drugs, disinfectants, and mechanical stress, making these biofilms a safety risk for the food industry [24, 25]. *Salmonella* biofilm capacity has been estimated at a laboratory scale on diverse surface materials [22, 23, 26].

There has been limited information about the molecular detections and biofilm profile of *Salmonella* serovars isolated from poultry. The *invA* gene encodes an essential component of the invasion-associated protein secretion apparatus, while the *spvC* gene promotes rapid growth and survival within the host [27]. The *sdiA* gene of *Salmonella* detects and responds to signals generated only by other microbial species [28]. Quorum-sensing genes have been used as targets in diagnostic PCR assays. In this study, *Salmonella* isolates recovered from poultry farms in Nigeria were analyzed for the presence of two virulence genes, *invA* and *spvC*, and a quorum sensing gene, *sdiA*. The objective of this study was to evaluate the prevalence, virulence and quorum sensing genes, multiple antibiotic resistance, phenotypic virulence factors and biofilm formation of *Salmonella* isolate recovered from poultry farms in Edo and Delta States, Nigeria.

## Materials and methods

### Study area

**Fig 1:**
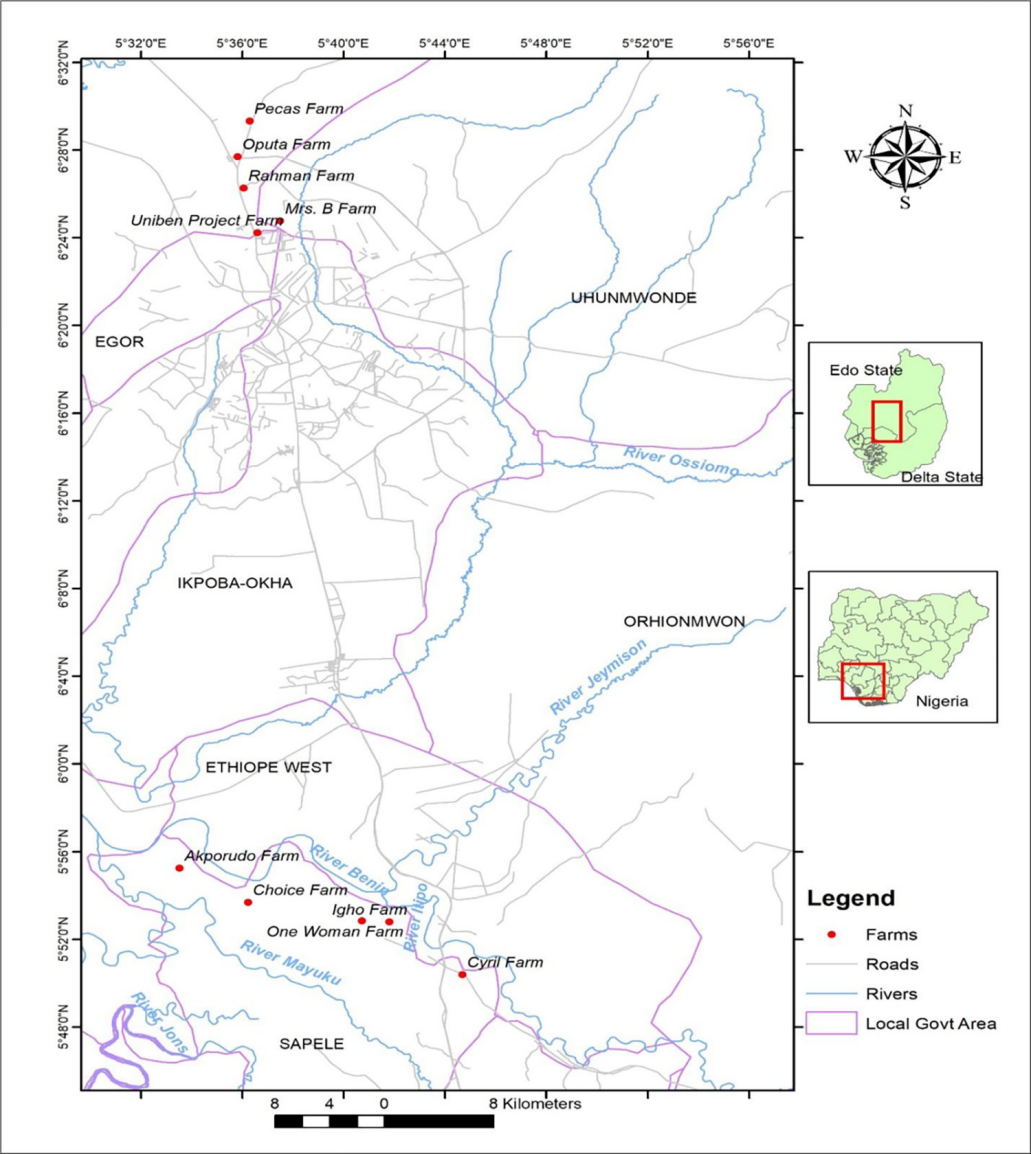
Map of the study area.

The samples were collected from ten farms in Edo and Delta States, Nigeria (5 farms each). The farms from Edo state include the UNIBEN Project farm, Oputa farm, Mrs B farm, Pecas farm and Rehmah farm. The farms from Delta State includes Ome Woman farm, Choice farm, Akporido farm, Igho farm, and Cyril farm. The locations of respective farms in Edo and Delta State are shown in Fig 1 below. The farms housed between 200–1,150 birds. The farms use the deep litter system or battery cage system.

In all poultry farms, birds are fed concentrate, pre-layer mash, layer phase 2, layer mash, broiler super starter, broiler finisher, broiler starter, grower mash, and chick mash from Top Feeds Nigeria and given water using water troughs. The poultry environment is cleaned once or twice daily, while antibiotics (such as amoxicillin, ampicillin, amprolium, kenflox, floxinor, gentamicin and erythromycin) are administered to the birds through their drinking water. Amin’total (combination of amino acids, vitamins and trace elements complex), vitawright (combinations of amino acids and vitamins), super three plus (a well-balanced multivitamin powder), mia vit VTA (high concentration of a wide range of vitamins, minerals and other trace elements) are administered to the birds through their drinking water. Dewormers such as kepromec, lavadex, interlava-200 ws and pipe dewormer wsp are administered to the birds via drinking water to treat gastrointestinal and lungworm infections. Tylo-dox extra (200 mg of doxycycline hyclate and 100 mg of tylosin tartrate) is given to the birds to treat respiratory and gastrointestinal diseases, and Neo-oxy (combination of neomycin and oxytetracycline) egg formula is given to the layers via their drinking water to boost egg production. A veterinary doctor also comes to check on the birds regularly.

### Ethical consideration

Ethical and study protocol approval for the research was obtained from the Research Ethics Review Board of the Faculty of Life Sciences (RERBLS), the University of Benin, Benin City, Nigeria, with reference number UNIBEN/RERBLS/103019 before sample collection. Verbal informed consent was obtained from all farm owners for inclusion before the sample collection.

### Sample collection

The sample size used in this study was determined using the sample size determination formula as follows:

$$\mathrm{Sample}(N)=\frac{{\left(Z_{1-\propto /2}\right)}^{2}P(1-P)}{d^{2}}$$

P = Expected prevalence based on the previous study [0.8% from Aragaw *et al*. [29]; 4.82% from Singh *et al*. [30] were used]; d = Absolute error or precision (which is 5%); Z_1-α/2_ = Standard normal variant at 5% type I error (P < 0.05). Hence, the expected sample size was ≤ 71 samples. A total of one hundred and fifty (150) samples were collected randomly from the ten different poultry farms in Delta and Edo States, Nigeria, between January 2020 and August 2020. The samples collected comprise 50 feed samples, 50 water samples, and 50 faecal samples. Sterile universal containers were used to collect water samples from the water trough, the feeds, and the faecal samples from the various farms. The samples were well-labelled with identification numbers and were transported immediately to the laboratory on ice for processing. The Guidelines performed in the study for the Care and Use of Agricultural Animals in Research and Teaching 3^rd^ ed. (http://www.fass.org/) [31] and the ethical guidelines of the Ethnic Research Committee of the University of Benin. All persons gave their verbal informed consent before including their birds in the study.

### Enrichment, isolation and phenotypic characterization

A stock solution was prepared by aseptically weighing 25.0g of faecal and feed samples into a sterile wide-mouth Erlenmeyer flask containing 225 mL tryptone soy broth (Lab M, United Kingdom); and the content was allowed to soak without homogenization; followed by incubation for 24 ± 2 h at 35°C. Similarly, 25 mL water samples were aseptically dispensed into 225 mL tryptone soy broth in a wide mouth, sterile, screw-capped jar; swirled thoroughly; left to stand at 28°C for 60 ± 5 min; followed by incubating the loosely capped container at 35°C for 24 ± 2 h. From both the solid and liquid incubated content, 0.1 mL was inoculated into 10 ml Rappaport-Vassiliadis medium (Merck, Germany); another 1 mL mixture was inoculated into 10 mL tetrathionate broth (Oxoid, UK). All contents were mixed thoroughly via the vortex. Rappaport-Vassiliadis (RV) medium was incubated at 42 ± 0.2°C for 24 ± 2 h. The tetrathionate (TT) broth was incubated at 43 ± 0.2°C for 24 ± 2 h. Both the RV and TT were incubated in a thermostatically controlled, circulating water bath. Both the RV and TT overnight content was streaked on xylose lysine desoxycholate (XLD) agar (Lab M, Lancashire, UK) and Hektoen enteric (HE) agar (Lab M, Lancashire, UK); and incubated at 35°C for 24 ± 2 h [32]. *Salmonella enterica* serovar Typhimurium ATCC 14028, and *Salmonella* Enteritidis ATCC 13076, were used as positive controls in all test procedures.

The plates were examined for colonies with glossy large black centres or almost black colonies on HE agar. For XLD agar, colonies with glossy large black centres or almost black colonies were examined. After that, distinct colonies per plate were picked and purified repeatedly on nutrient agar (Lab M, United Kingdom) plates. Pure isolates were stored on agar slants at 4°C for further analysis. The purified isolates obtained from the nutrient agar were subjected to the phenotypic characterization of 3% KOH for Gram reactions, catalase, oxidase, urease, and indole. Organisms that appear as Gram-negative rods and are catalase positive, oxidase negative, urease negative and indole negative were selected presumptively as *Salmonella*e [33]. According to the manufacturer’s instructions, colonies suspected of being *Salmonella* were confirmed using an API 20E kit (Biomérieux, l’Etoile, France).

### DNA extraction and 16S rRNA sequencing

The genomic DNA was extracted according to the method of Chen and Kuo [34]. The 27-F primer with the sequence 5’-AGAGTTTGATCMTGGCTCAG-3’ and the 1540-R primer with the sequence 5’-TACGGYTACCTTGTTACGACT-3’ were used for the amplification of the gene using PCR [35]. A 50 μL PCR mixture, consisting of 10 μL DNA (10ng μL^-1^), 5μL PCR buffer with MgCl_2_, 6μL dNTP mix, 2.5μL 27-F primer (10 pmol μL^-1^), 2.5μL 1540-R primer (10 pmol μL^-1^), 0.3μL Taq Polymerase and 23.7μL double distilled water (ddW) were used. There was an initial denaturation at 94°C for 3 min, followed by 32 cycles of denaturation at 94°C for the 30s, 30s of annealing at 56°C and 1min 30s of elongation at 72°C and final extension at 72°C for 5 min cycles. For gel electrophoresis, a 1.0% agarose gel was prepared composed of 4.0g agarose and 1×400 mL TAE buffer. After, 1.0μL GelRed (Merck KGaA, Darmstadt, Germany) was placed in the agarose gel before polymerising that per 100mL gel. The wells were filled with 5.0μL PCR products and 2.0μL DNA gel loading dye (Qiagen USA). The gel was run for one hour at a DC voltage of 100V. The 16S rRNA gene was successfully amplified when a DNA fragment at approximately 1500 bp could be recognized. The DNA purification kit Cycle-Pure Kit, peqGOLD, was used to purify the PCR products. For sequencing, the DNA concentration was measured using a microvolume spectrophotometer (NanoDrop, Thermofisher) and then adjusted to a concentration of between 20-80ng/μL. The sequences were compared with the database of the NCBI. By the "Basic Local Alignment Search Tool" (BLAST), the inserted 16S rRNA sequences were assigned to bacterial strains with identical or very similar 16S rRNA sequences. The isolates were also confirmed with a panel of primers [S1 Table in S1 File], and PCR conditions described previously [36]. *Salmonella enterica* serovar Typhimurium ATCC 14028, and *Salmonella* Enteritidis ATCC 13076, were used as positive controls in all test procedures. The DNA purification kit Cycle-Pure Kit, peqGOLD, was used to purify the PCR products.

### Antibiotics susceptibility testing

The susceptibility of the isolated bacteria to antibiotics was tested using the Kirby-Bauer disc diffusion method. A commercially available antibiotic disc obtained from Mast Diagnostics, Merseyside, United Kingdom, was used to determine the susceptibility patterns of the isolates as recommended by the Clinical and Laboratory Standards Institute [37]. The antibiotics used include piperacillin (PIP-100μg), ampicillin (AMP-10μg), gentamicin (GEN-10μg), amoxicillin/clavulanate (AMC-30μg), imipenem (IMI-10μg), meropenem (MEM-10μg), cefotaxime (CTX-30μg), sulfamethoxazole (SUL-25μg), azithromycin (AZI-15μg), chloramphenicol (CHL-30μg), ciprofloxacin (CIP-5μg) and tetracycline (TET-30μg). Pure cultures of identified bacteria were inoculated into 5.0 mL of Mueller Hinton broth (Lab M, United Kingdom) and incubated at 37°C for 24h. The inoculum was then spread on Mueller Hinton agar (Lab M, United Kingdom) using a sterile glass spreader. The antibiotics to be tested were placed aseptically onto the surface of the agar plates with sterile forceps and gently pressed to ensure even contact. The plates were incubated at 37°C for 18 to 24h (CLSI, 2018). The diameter of the zone of inhibition around each disc was measured and interpreted as resistance (R), sensitive (S) or intermediate (I) by the recommended standard established by CLSI to determine the intermediate, resistance and sensitivity profiles of the isolates to the antibiotics used. Multiple antibiotic resistance (MAR) was estimated using the formula MAR = a/b, where a represents the number of antibiotics the test isolate was resistant to, and b shows the total number of antibiotics tested [38].

### Detection of virulence genes

*Salmonella* isolates were screened by PCR methods to detect the occurrence of virulence genes (*spv*C and *inv*A). PCR amplification was carried out as described previously [39] using primer pairs presented in the S1 Table in S1 File. Amplification was carried out in a 25 μL final volume, with a reaction mixture which contains 5.0 μL green GO Taq buffer (5×); 1.0 μL bacterial DNA; 100 μM each deoxynucleoside triphosphates (dNTPs), 0.5U GO Taq DNA polymerase, and 0.125 μM of each primer. Amplification was conducted in the thermocycler. The PCR cycling of the virulence determinants *spv*C / *inv*A comprises an initial denaturation step (94°C for 4 min), followed by 40 cycles (94°C for the 30s). This is followed by annealing (52°C for the 30s), extension (72°C for 45s), and a final extension period (72°C for 7 min). PCR products (5μL) were visualized via electrophoresis in 1.5% (w v^-1^) agarose gels and viewed via UV transilluminator after staining with ethidium bromide. A molecular-weight DNA marker (100-bp DNA ladder) was used on the individual gel. *Salmonella* Typhimurium ATCC 14028 was used as the control for all reactions.

### Detection of quorum sensing genes

Primers *sdiA*2 and *sdiA*1 (S1 Table in S1 File) were used for *Salmonella* quorum sensing screening. Amplification was carried out in a final volume (25 μL) with a reaction mixture that contained 0.125 μM of respective *sdiA* primers [28]. The PCR cycling program of the *sdiA* gene comprised of initial denaturation (94°C for 5min), followed by 30 cycles of denaturation (94°C for the 30s), annealing (52°C for 40s), extension (72°C for 30s), and a final extension (72°C for 7min). PCR products (5μL) were visualized via electrophoresis in 1.5% (w v^-1^) agarose gels and viewed via UV transilluminator after staining with ethidium bromide. A molecular-weight DNA marker (100-bp DNA ladder) was used on the individual gel. *Salmonella* Typhimurium ATCC 14028 was used as the control for all reactions.

### Evaluation of extracellular virulence factors

Colonies cultivated on tryptone soy agar (TSA) were suspended in 3mL of Mueller Hinton broth. The density of this suspension was adjusted to 0.5 McFarland standards, the equivalent of 10^8^ cells μL^-1^. An aliquot of 0.5 mL sample of this suspension was used in each extracellular virulence enzyme assay and incubated for 24 to 48 h at 37°C. Extracellular protease activity of the isolates was determined on TSA plates supplemented with 1% casein (v v^-1^). The zone of clearance due to casein hydrolysis was considered a positive result [40]. The lipase activity was determined on TSA plates supplemented with 1% Tween 80 (v v^-1^). A clear halo surrounds the areas where the lipase-producing organism has proliferated [41]. The β-haemolytic activity was carried out on sheep blood agar plate and incubated for 24 to 48 h at 37°C. β-haemolysis was evaluated by clear colourless zones surrounding the colonies, indicating that there has been total lysis of the red blood cells. The gelatinase production was assayed in a gelatin medium (5% peptone, 3% beef extracts, pH 7.0, 15% gelatin). A zone of clearance in the media reveals the presence of a gelatin-liquefying bacterium [26]. DNA degrading activity was assayed on DNase agar plates. When DNA is degraded, methyl green is released, turning the medium colourless around the test organism [40].

### Characterization of biofilm-forming capacity

*Salmonella*’s biofilm formations were screened using the microtiter plate method described previously [42]. A 96-well microtiter plate was dispensed with 200μL TSB and inoculated with 20μL of *Salmonella* isolates, grown overnight and standardized to 0.5 McFarland standards. This was followed by incubation for 48h at 37°C. The constituents of individual wells were removed and washed 3× with sterile phosphate-buffered saline (PBS); left to dry, and stained for 30min with 200μL of 1% crystal violet. The wells were carefully washed 3× with distilled H_2_O to remove the excess dye and allowed dry at 30°C. Adherent cells that are dye-bound were resolubilized with 150μL of ethanol. The plates were then read with a microplate reader (Synergy MX Biotek, USA) at 570nm wavelength. The independent biological duplicate’s average optical density (OD) was taken into positive and negative controls. The isolates were categorized as strong (ODi> 0.12), moderate (ODi = 0.1 < 0.12), weak (ODc<ODi<0.1), and non-biofilm (ODi<ODc) producers [43].

## Results

### *Salmonella* occurrence in the samples

The distribution of the total *Salmonella* occurrence in samples from poultry farms in Edo and Delta States, based on the proliferation of black colonies on xylose lysine deoxycholate agar, is presented in Table 1. The occurrence of *Salmonella* in the total number of samples investigated in Edo State was 3/20 for the faecal samples, while the feed and water samples had no *Salmonella* growth. The distribution of total *Salmonella* recorded in representatives from Delta State was 7/30 and 1/30 for faecal and water samples, respectively, while there was no *Salmonella* recorded for feed samples. Sequenced *Salmonella* isolates had a percentage similarity between 98–100% and were identical when subjected to specific primer sets. The isolates that were neither *Salmonella* Enteritidis nor *Salmonella* Typhimurium were grouped as other *Salmonella* species. *Salmonella* serovars identified include: *Salmonella* Enteritidis [*n* = 17 (39.5%)], *Salmonella* Typhimurium [*n* = 13 (30.2%)] and other *Salmonella* serovars [*n* = 13 (30.2%)].

**Table 1:** *Salmonella* occurrence from samples in the poultry farms in Edo and Delta State.

	Samples	The total number of samples investigated	Total number of *Salmonella-positive* samples	Percentage (%) number of *Salmonella* positive samples
Edo State	Faecal	20	3	15%
	Feed	20	0	0%
	Water	20	0	0%
Delta State	Faecal	30	7	23.33%
	Feed	30	0	0%
	Water	30	1	3.33%

**Fig 2:**
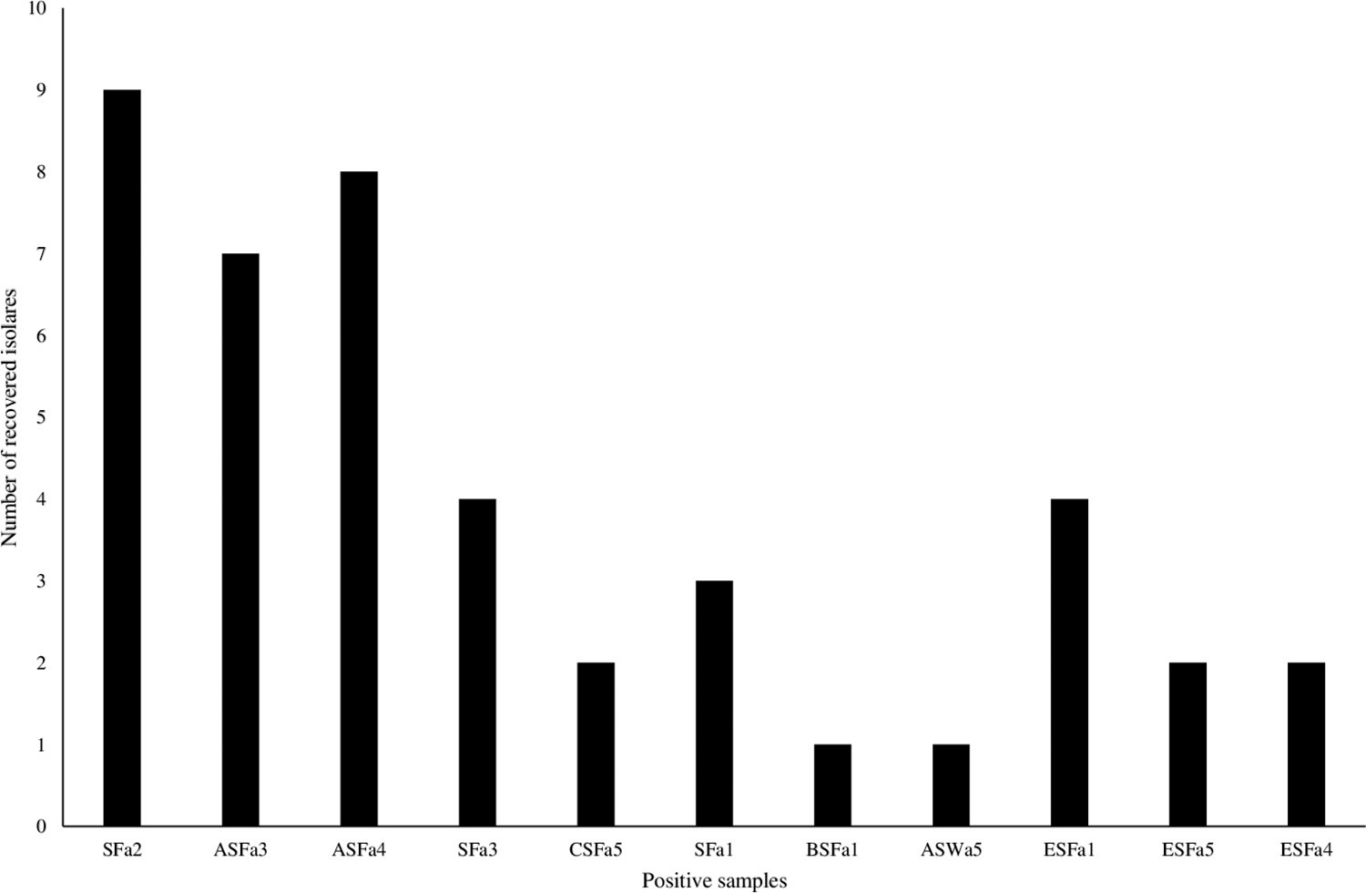
Frequency of *Salmonella* isolated from positive samples.

A total of 43 *Salmonella* serovars were found in 11 samples (10 faecal samples and one water sample) (Fig 2). The ten faecal samples were coded SFa2, ASFa3, ASFa4, SFa3, CSFa5, SFa1, BSFa1, ESFa1, ESFa5 and ESFa4; while ASWa5 was a water sample (Fig 2). A total of 11/150(7.33%) samples were positive for *Salmonella* serovars. The prevalence based on the sample includes: faecal 10/50, and water 1/50, while none of the 50 feed samples had *Salmonella*.

A total of 6/10farms were positive for *Salmonella* serovar (S2 Table in S1 File). The distribution of *Salmonella* serovars based on farms sampled includes Mrs B farm [*Salmonella* Enteritidis (6), *Salmonella* Typhimurium (2), other *Salmonella* serovars (1)]; One Woman farm [*Salmonella* Enteritidis (4), *Salmonella* Typhimurium (4), different *Salmonella* serovars (7)]; Pecas farm [*Salmonella* Enteritidis (2), *Salmonella* Typhimurium (2), other *Salmonella* serovars (1)]; Akporido farm [*Salmonella* Enteritidis (1), *Salmonella* Typhimurium (1)]; Oputa farm [*Salmonella* Enteritidis (2), *Salmonella* Typhimurium (1)]; Cyril farm [*Salmonella* Enteritidis (2), *Salmonella* Typhimurium (3), other *Salmonella* serovars (4)]. The only water sample with *Salmonella* was from One Woman farm with isolate code (OSS_33_), while the different positive samples were from faecal sources. There is no connection between certain farms and a particularly high number of positive samples (S2 Table in S1 File).

### Antimicrobial susceptibility profile of *Salmonella* serovars

The antimicrobial susceptibility profile of the *Salmonella* isolates is presented in Table 2. Findings from this study revealed a high level of resistance of *Salmonella* Enteritidis to the following antibiotics: piperacillin and ampicillin (17, 100%); amoxicillin/clavulanate (14, 82.35%); cefotaxime (17, 100%); and ciprofloxacin (15, 88.23%) and they showed a high level of sensitivity to gentamicin and chloramphenicol (14, 82.35%); tetracycline (13, 76.47%); imipenem, meropenem (17, 100%). *Salmonella* Typhimurium revealed a high level of resistance to piperacillin and ampicillin (13, 100%); amoxicillin/clavulanate (9, 69.23%); cefotaxime (13, 100%); azithromycin (9, 69.23%); and ciprofloxacin (11, 84.62%) and were susceptible to gentamicin (13, 100%); sulfamethoxazole (11, 84.62%); chloramphenicol (9, 69.23%); tetracycline (8, 61.54%); imipenem, meropenem (13, 100%). Other *Salmonella* serovars revealed a high level of resistance to piperacillin (12, 92.31%); ampicillin, cefotaxime (13, 100%); azithromycin (8, 61.54%); ciprofloxacin (12, 92.31%); and high level of sensitivity to gentamicin (11, 84.62%); sulfamethoxazole and chloramphenicol (8, 61.54%); tetracycline (7, 53.85%); and imipenem, meropenem (13, 100%).

**Table 2:** Antimicrobial susceptibility profiles of the *Salmonella* serovars.

Antimicrobial	Antibiotics (disc content)	Salmonella Enteritidis			Salmonella Typhimurium			Other Salmonella serovars		
		(n = 17)			(n = 13)			(n = 13)		
Class										
		R (%)	I (%)	S (%)	R (%)	I (%)	S (%)	R (%)	I (%)	S (%)
Penicillin	Piperacillin	17(100)	0	0	13(100)	0	0(0)	12(92.31)	1(7.69)	0
Penicillin	Ampicillin	17(100)	0	0	13(100)	0	0	13(100)	0	0
Aminoglycosides	Gentamicin	1(5.88%)	2(11.76)	14(82.35)	0(0)	0	13(100)	1(7.69)	1(7.69)	11(84.62)
Β-lactam/	Amoxicillin/	14(82.35)	1(5.88%)	2(11.76)	9(69. 23)	1(7.69)	3(23.08)	8(61.54)	2(15.38)	3(23.08)
β-lactamase	Clavulanate									
Inhibitors										
Carbapenems	Imipenem	0	0	17(100)	0	0	13(100)	0	0	13(100)
	Meropenem	0	0	17(100)	0	0	13(100)	0	0	13(100)
Cephalosporins	Cefotaxime	17(100)	0	0	13(100)	0	0	13(100)	0	0
Folate pathway	Sulfamethoxazole	7(41.18)	3(17.65)	7(41.18)	2(15.38)	0	11(84.62)	1(7.69)	4(30.77)	8(61.54)
Inhibitors										
Macrolides	Azithromycin	9(52.94)	0	8(47.06)	9(69.23)	0	4(30.77)	8(61.54)	0	5(38.46)
Phenicols	Chloramphenicol	2(11.76)	1(5.88)	14(82.35)	1(7.69)	3(23.08)	9(69.23)	2(15.38)	3(23.08)	8(61.54)
Quinolone	Ciprofloxacin	15(88.23)	2(11.76)	0	11(84.62)	2(15.38)	0	12(92.31)	1(7.69)	0
Tetracyclines	Tetracycline	4(23.53)	0	13(76.47)	3(23.08)	2(15.38)	8(61.54)	2(15.38)	4(30.77)	7(53.85)

R: Resistance

I: Intermediate

S: Sensitive

### Multiple antibiotics resistance profile

The multiple antibiotics resistance profile of the *Salmonella* isolates is presented in Table 3. A total of 3 (17.65%) *Salmonella* Enteritidis were resistant to 5 antibiotics (PIP^R^, AMP^R^, AMC^R^, CTX^R^, CIP^R^) which belong to 4 antimicrobial classes with a multiple antibiotics resistance index (MARI) of 0.42. A total of 2 (11.76%) each were resistant to 6 antibiotics (PIP^R^, AMP^R^, AMC^R^, CTX^R^, AZI^R^, CIP^R^) and (PIP^R^, AMP^R^, AMC^R^, CTX^R^, CIP^R^, TET^R^) which belongs to 5 antimicrobial class each with a MARI of 0.5 each. A total of 3 (23.08%) of *Salmonella* Typhimurium were resistant to 6 antibiotics (PIP^R^, AMP^R^, AMC^R^, CTX^R^, AZI^R^, CIP^R^) which belong to 5 antimicrobial classes with a MARI of 0.5. A total of 2 (15.38%) each were resistant to 5 antibiotics (PIP^R^, AMP^R^, CTX^R^, AZI^R^, CIP^R^) and (PIP^R^, AMP^R^, AMC^R^, CTX^R^, CIP^R^) which belong to 4 antimicrobial class each with a MARI of 0.42 each. A total of 3 (23.08%) of other *Salmonella* serovars were resistant to 5 antibiotics (PIP^R^, AMP^R^, CTX^R^, AZI^R^, CIP^R^) which belong to 4 antimicrobial classes with a MARI of 0.42. A total of 2 (15.38%) were resistant to 6 antibiotics (PIP^R^, AMP^R^, AMC^R^, CTX^R^, CHL^R^, CIP^R^) which belongs to 5 antimicrobial class with a MARI of 0.5. Furthermore, all the *Salmonella* serovars showed resistance to 2 antibiotics (AMP^R^, CTX^R^).

**Table 3:** Multiple antibiotics-resistant distribution of the *Salmonella* isolates.

Salmonella serovars	Isolate codes	No antimicrobial class	No antibiotics	Resistance phenotypes	Salmonella serovars (%)	MARI
*Salmonella* Enteritidis (*n* = 17)	SE_13_, SE_20_, SE_21_	4	5	PIP^R^, AMP^R^, AMC^R^, CTX^R^, CIP^R^	3 (17.65)	0.42
	SE_3_, SE_22_	5	6	PIP^R^, AMP^R^, AMC^R^, CTX^R^, AZI^R^, CIP^R^	2(11.76)	0.50
	SE_15_, SE_17_	5	6	PIP^R^, AMP^R^, AMC^R^, CTX^R^, CIP^R^, TET^R^	2 (11.76)	0.50
	SE_1_	8	9	PIP^R^, AMP^R^, GEN^R^, AMC^R^, CTX^R^, SXT^R^, CHL^R^, AZI^R^, CIP^R^	1 (5.88)	0.75
	SE_2_	6	7	PIP^R^, AMP^R^, AMC^R^, CTX^R^, SXT^R^, AZI^R^, CIP^R^	1 (5.88)	0.58
	SE_5_	5	6	PIP^R^, AMP^R^, AMC^R^, CTX^R^, SXT^R^, CIP^R^	1 (5.88)	0.50
	SE_16_	6	7	PIP^R^, AMP^R^, AMC^R^, CTX^R^, AZI^R^, CIP^R^, TET^R^	1 (5.88)	0.58
	SE_18_	7	8	PIP^R^, AMP^R^, AMC^R^, CTX^R^, SXT^R^, AZI^R^, CIP^R^, TET^R^	1 (5.88)	0.67
	SE_25_	3	4	PIP^R^, AMP^R^, AMC^R^, CTX^R^	1 (5.88)	0.5
	SE_26_	7	8	PIP^R^, AMP^R^, AMC^R^, CTX^R^, SXT^R^, CHL^R^, AZI^R^, CIP^R^	1 (5.88)	0.67
	SE_35_	3	4	PIP^R^, AMP^R^, CTX^R^, AZI^R^	1 (5.88)	0.33
	SE_36_	4	5	PIP^R^, AMP^R^, CTX^R^, SXT^R^, CIP^R^	1 (5.88)	0.42
	SE_37_	5	6	PIP^R^, AMP^R^, CTX^R^, SXT^R^, AZI^R^, CIP^R^	1 (5.88)	0.50
*Salmonella* Typhimurium (*n* = 13)	ST_10_, ST_11_, ST_24_	5	6	PIP^R^, AMP^R^, AMC^R^, CTX^R^, AZI^R^, CIP^R^	3 (23.08)	0.50
	ST_32_, ST_39_	4	5	PIP^R^, AMP^R^, CTX^R^, AZI^R^, CIP^R^	2 (15. 38)	0.42
	ST_14_, ST_19_	4	5	PIP^R^, AMP^R^, AMC^R^, CTX^R^, CIP^R^	2 (15.38)	0.42
	ST_27_	6	7	PIP^R^, AMP^R^, AMC^R^, CTX^R^, AZI^R^, CIP^R^, TET^R^	1 (7.69)	0.58
	ST_28_	5	6	PIP^R^, AMP^R^, AMC^R^, CTX^R^, CIP^R^, TET^R^	1 (7.69)	0.50
	ST_30_	4	5	PIP^R^, AMP^R^, AMC^R^, CTX^R^, AZI^R^	1 (7.69)	0.42
	ST_31_	4	5	PIP^R^, AMP^R^, AMC^R^, CTX^R^, SXT^R^	1 (7.69)	0.42
	ST_38_	5	6	PIP^R^, AMP^R^, CTX^R^, CHL^R^, AZI^R^, CIP^R^	1 (7.69)	0.50
	ST_43_	6	7	PIP^R^, AMP^R^, CTX^R^, SXT^R^, AZI^R^, CIP^R^, TET^R^	1 (7.69)	0.58
Other *Salmonella* serovars (*n* = 13)	OSS_33_, OSS_34_, OSS_41_	4	5	PIP^R^, AMP^R^, CTX^R^, AZI^R^, CIP^R^	3 (23.08)	0.42
	OSS_6_, OSS_9_	5	6	PIP^R^, AMP^R^, AMC^R^, CTX^R^, CHL^R^, CIP^R^	2 (15.38)	0.50
	OSS_4_	5	6	PIP^R^, AMP^R^, GEN^R^, AMC^R^, CTX^R^, CIP^R^	1 (7.69)	0.50
	OSS_7_	4	5	PIP^R^, AMP^R^, AMC^R^, CTX^R^, CIP^R^	1 (7.69)	0.42
	OSS_8_	5	6	PIP^R^, AMP^R^, AMC^R^, CTX^R^, AZI^R^, CIP^R^	1 (7.69)	0.50
	OSS_12_	4	5	PIP^R^, AMP^R^, AMC^R^, CTX^R^, AZI^R^	1 (7.69)	0.42
	OSS_23_	5	5	AMP^R^, AMC^R^, CTX^R^, AZI^R^, CIP^R^	1 (7.69)	0.42
	OSS_29_	6	7	PIP^R^, AMP^R^, AMC^R^, CTX^R^, AZI^R^, CIP^R^, TET^R^	1 (7.69)	0.58
	OSS_40_	6	7	PIP^R^, AMP^R^, AMC^R^, CTX^R^, AZI^R^, CIP^R^, TET^R^	1 (7.69)	0.58
	OSS_42_	3	4	PIP^R^, AMP^R^, CTX^R^, CIP^R^	1 (7.69)	0.33

MARI: Multiple Antibiotic Resistance Index

SE = *Salmonella* Enteritidis, ST = *Salmonella* Typhimurium, OSS = Other *Salmonella* serovars, piperacillin (PIP-100μg), ampicillin (AMP-10μg), gentamicin (GEN-10μg), amoxicillin/clavulanate (AMC-30μg), imipenem (IMI-10μg), meropenem (MEM-10μg), cefotaxime (CTX-30μg), sulfamethoxazole (SUL-25μg), azithromycin (AZI-15μg), chloramphenicol (CHL-30μg), ciprofloxacin (CIP-5μg) and tetracycline (TET-30μg).

### Distribution of the virulence and quorum sensing gene

The distribution of the virulence (*invA* and *spvC*) genes and the quorum-sensing (*sdiA*) gene is shown in Table 4. Of the 17 *Salmonella* Enteritidis, 13 *Salmonella* Typhimurium and 13 other *Salmonella* serovars, 15(88.24%) and 10(58.82%) of *Salmonella* Enteritidis, 13(100%) and 4(30.77%) of *Salmonella* Typhimurium, 9(69.23%) and 2(15.38%) of the other *Salmonella* serovars were positive for the virulence *invA* and *spvC* genes respectively. Furthermore, all the *Salmonella* serovars; 17(100%), 13(100%) and 13(100%) of *Salmonella* Enteritidis, *Salmonella* Typhimurium and other *Salmonella* serovars, respectively, were positive for the quorum sensing *sdiA* gene.

**Table 4:** Distribution of virulence and quorum sensing genes.

*Salmonella* isolates (*n* = 43)	Virulence genes		Quorum sensing gene
	*invA*	*spvC*	*SdiA*
*Salmonella* Enteritidis (*n* = 17)	15(88.24)	10(58.82)	17(100)
*Salmonella* Typhimurium (*n* = 13)	13(100)	4(30.77)	13(100)
Other *Salmonella* serovars (*n* = 13)	9(69.23)	2(15.38)	13(100)

Values in parenthesis represent percentage (%)

### Extracellular virulence factors of the *Salmonella* serovars

**Fig 3:**
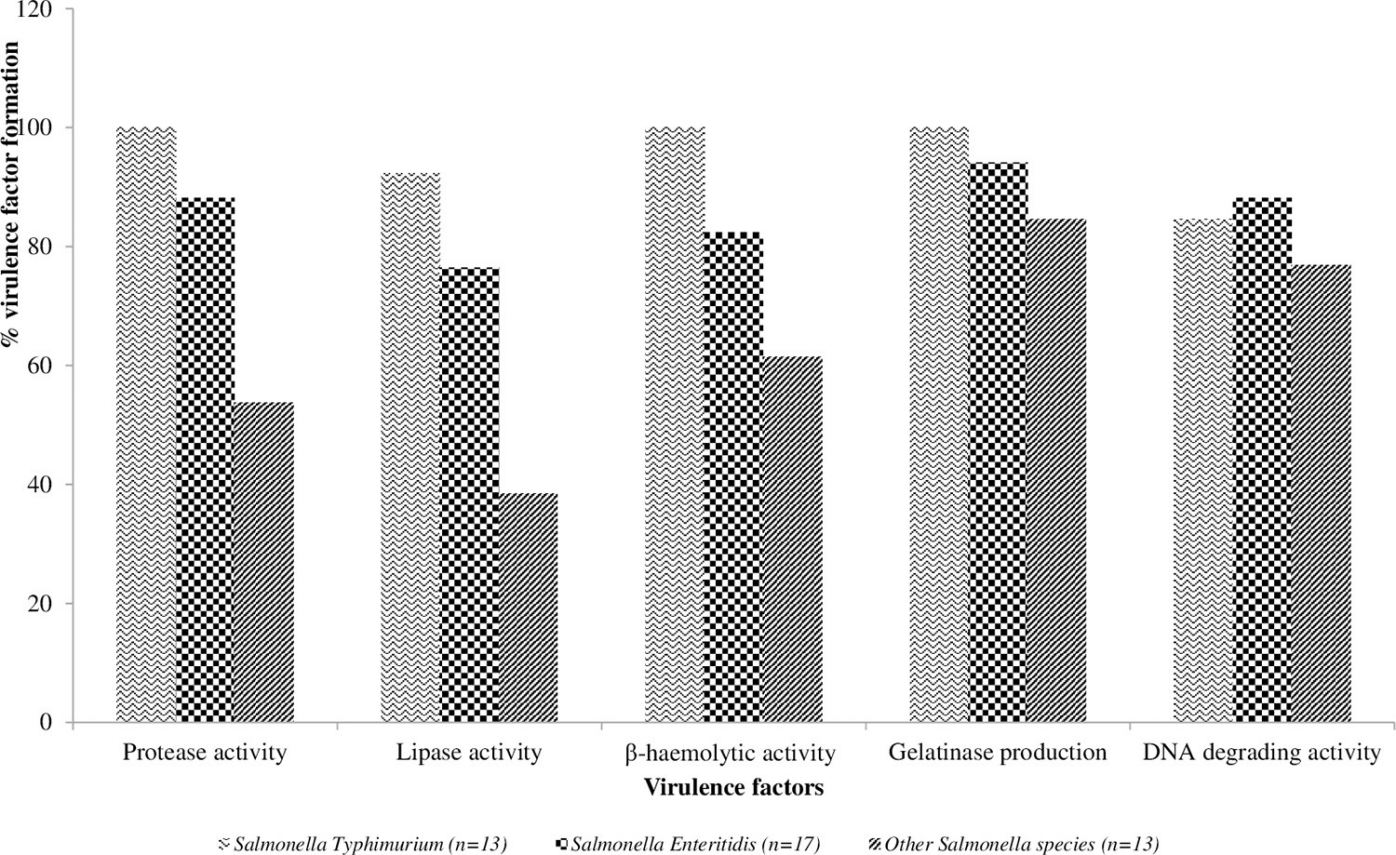
Distribution of phenotypic virulence factors of the *Salmonella* serovars.

Fig 3 shows the distribution of the phenotypic virulence factors of the *Salmonella* isolates. A total of 15(88.23%), 13(76.47%), 14(82.35%), 16(94.12%) and 15(88.24%) of the *Salmonella* Enteritidis isolates showed protease activity, lipase activity, β-hemolytic activity, gelatinase production and DNA degrading activity respectively. In addition, 13(100%) of the *Salmonella* Typhimurium isolates showed protease activity, β-hemolytic activity and gelatinase production, while 12(92.31%) and 11(84.62%) of the *Salmonella* Typhimurium isolates showed lipase activity and DNA degrading activity, respectively. For the other *Salmonella* serovars, 7(53.85%), 5(38.46%), 8(61.54%), 11(84.62%) and 10(76.92%) showed protease activity, lipase activity, β-hemolytic activity, gelatinase production and DNA degrading activity respectively.

### Biofilm profile of *Salmonella* serovars

**Fig 4:**
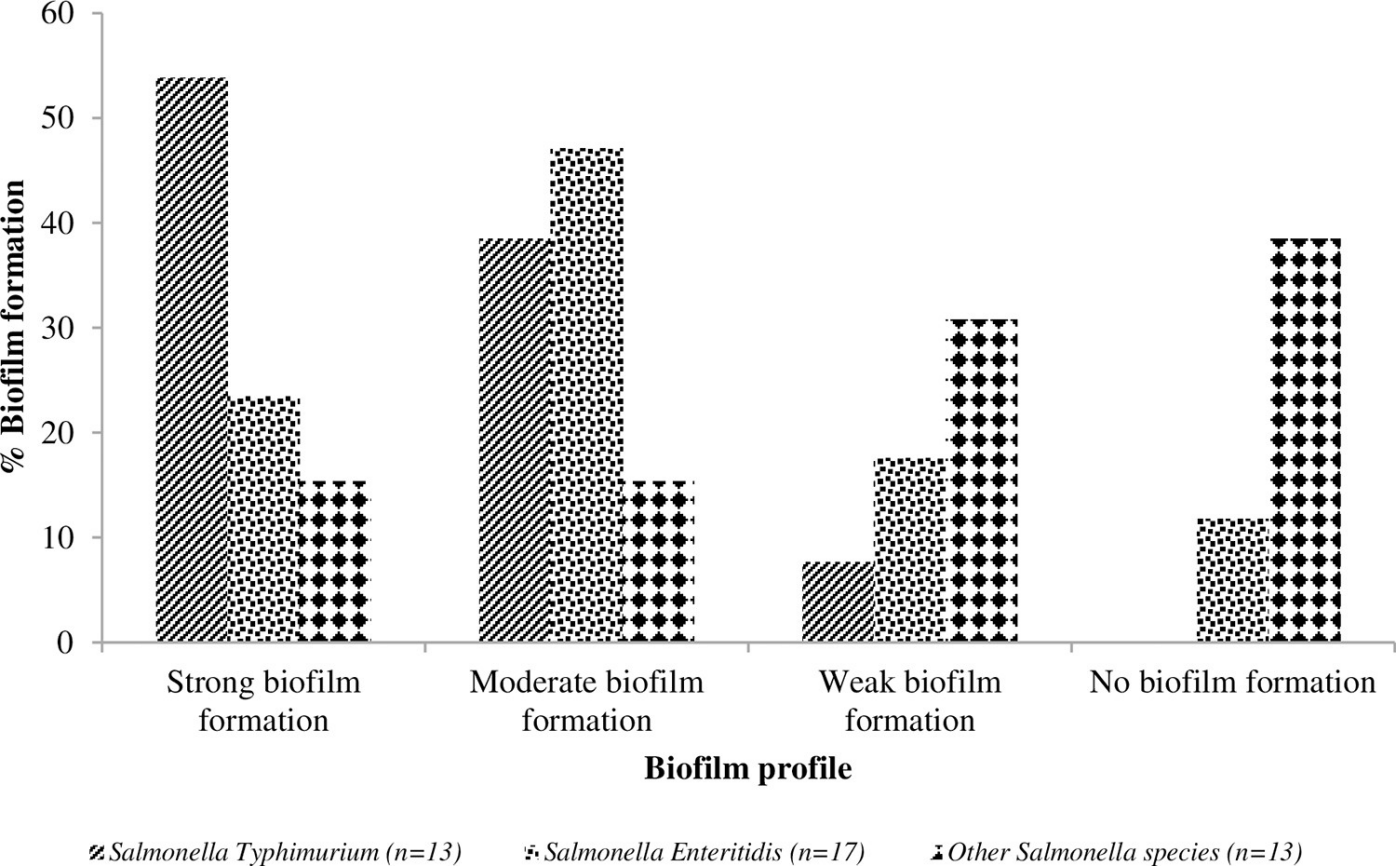
Biofilm formation profile of the *Salmonella* serovars.

Fig 4 shows the biofilm profile of the *Salmonella* isolates. For *Salmonella* Enteritidis isolates, 4(23.53%), 8(47.06%), 3(17.65%) and 2(11.76%) showed strong, moderate, weak, and no biofilm formation, respectively. In addition, 7(53.85%), 5(38.46%), and 1(7.69%) showed strong, moderate, and weak biofilm formation, respectively for the *Salmonella* Typhimurium isolates. Furthermore, 2(15.38%) of the other *Salmonella* serovars showed strong and moderate biofilm formation, while 4(30.77%) and 5(38.46%) showed weak and no biofilm formation, respectively.

## Discussion

The prevalence of *Salmonella* in poultry is a serious food safety issue as *Salmonella* pathogens can be transferred to humans via poultry products like meat and eggs [23, 30, 44]. Therefore, it has become essential that foodborne salmonellosis is constantly monitored. This study investigated Salmonella serovars’ occurrence, distribution and AMR from poultry farms in Edo and Delta States, Nigeria. This study revealed that some of the farms were susceptible to *Salmonella* contamination which was apparent in the *Salmonella* occurrence in the faecal and water samples. Higher *Salmonella* prevalence compared to findings from our study has been reported by Zishiri *et al*. [45] (51%) from South Africa and Brazil. Another study by Im *et al*. [46] also reported a higher prevalence (59.3%) from Korea. Other studies have also reported higher prevalence [47, 48] predominantly from faecal samples. A high *Salmonella* recovery rate from faecal samples has also been documented in Malaysia [49]. The high recovery rate from these studies may be due to sampling or environmental features such as seasonal variations [50, 51]. The feed, housing, and hygiene status of the poultry farms have been reported also contribute to *Salmonella* prevalence [52]. The difference in *Salmonella* prevalence could be attributed to the difference in the regions, seasons, sample types, sample sizes, isolation methods, culture technique, culture media, and environmental factors [50, 51, 53].

Feed and water consumed by farm animals have also been implicated as a reservoir of *Salmonella* in animal farms [54]. Egbule [55] reported 29% *Salmonella* occurrence from poultry feeds in Nigeria, which was high as no feeds from our study harboured *Salmonella* serovars. A survey from Iraq [56] reported that all feed samples were free of *Salmonella* similar to ours. This study revealed 1.11% (*n* = 1) of *Salmonella*, which was not closely identical to Bhatta *et al*. [39] (14%) and Osman et al. [49] (14.3%) prevalence rates of *Salmonella* revealed from water samples. The various possible factors that could result in the low occurrence of *Salmonella* isolates in this study were the proper handling of the bird feeds, good water quality, treatment of birds, hygienic measures observed in the poultry, and the regular check-up by the veterinary doctors [57]. The low occurrence of *Salmonella* in the poultry farms in this study may show low potential for the pathogen to disseminate from the farms to communities.

*Salmonella* infections significantly cause invasive and focal infections, and this ability varies with serovars [58]. This is closely in agreement with some studies [25, 59] with contrary findings reported by Bhatta *et al*. [39]. El-Sharkawy *et al*. [47] and Lamas *et al*. [50] said that *Salmonella* Enteritidis were not the most frequently recovered isolate of *Salmonella* serovar. The most commonly detected *Salmonella* serovars by Kim et al. [59] were *Salmonella* London (22.2%), *Salmonella* Albany (21.6%), *Salmonella* Bareilly (17.0%), and *Salmonella* Indiana (16.5%) which was not similar to the findings from our study.

Other studies revealed their dominant serovars as; *Salmonella* Tennessee [60] and *Salmonella* Enteritidis [61]. This difference in predominant serovars is attributed to pathogenicity, the adaptation of serovars to specific hosts, host specificity, geographical region, and diversities [5]. Alemu and Zewde [62] reported *Salmonella* Typhimurium and *Salmonella* Enteritidis as the most commonly associated serovars with food products and are the primary cause of salmonellosis in humans worldwide. Previous reports from Borges *et al*. [61], Gad *et al*. [60], and Lamas *et al*. [50] have observed the prevalence of other serovars such as *Salmonella* Anatum, *Salmonella* Tennessee, *Salmonella* Infantis and *Salmonella* Seftenberg. Human infections with *Salmonella enterica* serovar Enteritidis have been connected to egg products and egg consumption [44]. Implementation of cleaning and disinfection procedures, rodent control, and metal house walls significantly lowered the prevalence of *Salmonella* [59].

According to Igbinosa *et al*. [63], it has become common knowledge that antibiotics are applied in animal husbandry to treat, prevent bacterial infection, and enhance growth. They are also used for prevention and therapeutic purposes during outbreaks. *Salmonella* infections are generally self-limiting; however, antimicrobial therapy is used in cases where symptoms persist [64]. Most of the *Salmonella* isolates in this study revealed a higher resistance phenotype than a previous study from Malaysia [49]. This finding agreed with the Igbinosa *et al*. [63], where *Salmonella* Enteritidis and *Salmonella* Typhimurium and other serovars of *Salmonella* were resistant to various antibiotics. Similar findings were reported by Ahmed and Shimamoto [65], where it was reported that *Salmonella* Enteritidis and *Salmonella* Typhimurium serovars are resistant to ampicillin, amoxicillin/clavulanate, cefotaxime, and ciprofloxacin. Also, similar findings were reported by Zishiri *et al*. [45] and Beshiru *et al*. [25]. All isolates by Obe et al. [66] were resistant to multiple antibiotics, similar to our findings. In addition, 64% of Obe et al. [66] isolates exhibited resistance to aminoglycosides and beta-lactams, which was way higher than our study’s. A lower MDR of 52.3% compared to our study was reported previously [67]. High-level resistance has also been documented [56, 68, 69]. This poses a severe public health threat due to the resistance to a broad range of antibiotics observed in this study and could be a consequence of continuous exposure and extensive usage of these antibiotics in these study areas [27].

The isolates revealed intermediate resistance to tetracycline, chloramphenicol, gentamicin and trimethoprim-sulfamethoxazole, which is in line with the findings of Beshiru *et al*. [25] and Igbinosa [42]. Interestingly, few *Salmonella* serovars from our study were resistant to gentamicin, similar to the previous study [16]. This is interesting because gentamicin antibiotics in Nigeria are not sold as oral drugs but administered as injections. Hence, abuse or misuse of this particular antibiotic could be difficult as injections are usually based on the doctor’s prescriptions. However, since gentamicin shares the same antimicrobial group with kanamycin and streptomycin, which are often sold as capsules or caplets, the tendency of resistance is enhanced based on their target sites [70]. Kim et al. [59] reported that *Salmonella* Enteritidis isolates were resistant to ≥12 antibiotics, including third-generation cephalosporins and gentamicin. This is a serious concern because third-generation cephalosporins are critical antibiotics for the treatment of salmonellosis.

Multidrug resistance poses a serious threat to humans and animals and is of public health menace [38, 71]. The *Salmonella* serovars in this study revealed a high level of multidrug resistance as classified previously [72]. This study also corroborates results presented by Zhao *et al*. [73], where *Salmonella* Enteritidis and *Salmonella* Typhimurium have been reportedly linked with multidrug-resistant phenotypes. All *Salmonella* Enteritidis isolates by Kim et al. [59] were multidrug resistant, higher than our study. Previous studies have shown that infections due to multidrug-resistant *Salmonella* serovars show more adverse effects than those from sensitive strains as they delay therapy, further endangering patients’ lives [74]. All isolates by Siddique et al. [24] showed multiple drug resistance and were found to exhibit a high multiple antibiotic-resistant (MAR) index of 0.62 to 0.91, which was higher when compared to our study. The antibiotic-resistant elements in *Salmonella* serovars have made it more problematic due to the environment’s horizontal spread of resistant genes [75]. The occurrence and transfer of resistant elements of pathogenic bacteria, including gene dissemination in human intestinal microbiota, have been reported [76].

Findings from our study on the *invA* and *spvC* genes from the *Salmonella* isolates are closely in line with Borges et al. [61], which revealed that 100% of *Salmonella* Enteritidis and *Salmonella* Typhimurium were positive for the *invA* gene. In comparison, 91.4% and 12.5% of *Salmonella* Enteritidis and *Salmonella* Typhimurium, respectively, were positive for the *spvC* gene. This is also similar to the result of Lamas *et al*. [50] and Halatsi *et al*. [28], where it was revealed that all the *Salmonella* serovars were *invA* gene-positive which is crucial to *Salmonella* with the prevalence of a highly conserved DNA sequence for *Salmonella* detection [77] and to enter the host to cause infection thus upsurging the virulence of the isolates [25]. This study showed the presence of quorum sensing (QS) *sdiA* genes in all the *Salmonella* isolates. This is similar to the findings of Halatsi *et al*. [28], which revealed that all the *Salmonella* isolates were QS *sdiA* gene positive. QS is described as a communication mechanism that exists between bacteria, and it allows the control of specific processes, such as extracellular virulence factor formation, formation of biofilm, secondary metabolites production, as well as mechanisms that aid stress adaptation (such as bacterial systems that enhances competition with secretion systems inclusive) [78]. Molecular characterization by Obe et al. [66] showed that the isolates possessed specific genes for biofilm formation.

Our data showed that the *Salmonella* serovars from poultry had extracellular virulence characteristics. These external virulence factors were hemolysis, lipase, gelatinase, the presence of protease, and DNA degrading activity. A previous study reported a significant linkage between protease production, surface adherence, and pathogenesis [24, 26]. Extracellular protease, DNA structure, and lipolytic activity have been reported to correlate positively with biofilm formation [24, 26, 40]. The phenotypic virulence properties of 95 *Salmonella* isolates by Siddique et al. [24] from Pakistan exhibited DNA degrading activity 93(97.8%), hemolytic activity 92(96.8%), lipase activity 87(91.6%), and protease activity 86(90.5%) which were similar to the findings from our study.

The relationship between biofilm formation, which is keenly linked with QS, and secretion systems, has been demonstrated [79]. Biofilms have been associated with many outbreaks of pathogens and up to 80% of microbial infections [80]. Therefore, this study demonstrated the biofilm-forming ability of *Salmonella* Enteritidis isolates. The results by Ashrafudoulla et al. [22] indicated that the virulence factors and practical biofilm-forming ability of *Salmonella* Enteritidis isolates could affect human health and economic revenue. Previous studies have revealed that *Salmonella* serovars can form biofilms on different surfaces [22, 40]. Similar findings of biofilm formation were reported by Igbinosa [42], where strong, moderate and weak biofilm formations of *Salmonella* serovars were revealed. The isolates of Obe et al. [66] possessed strong (24%), moderate (28%), and weak (48%) biofilm-forming abilities. *Salmonella* has been reported as biofilm former at the minimal nutrient level [40]. *Salmonella* can adhere and form biofilms in water distribution pipelines [81]. In addition, water distribution systems have promoted conditions for developing the biofilm community [39]. Biofilm enhances their survival and persistence while increasing their chances of transmission to other animals, feed, water and humans. Bacteria grown in biofilms more remarkable transfer genes horizontally more than planktonic cells [61]. Biofilms increase the chances of gene transfer with the help of virulence factors and antibiotic-resistant genes from resistant to susceptible bacterial species, which leads to the emergence of new antibiotic resistance in pathogens [24].

## Conclusions

The results indicated that the poultry environment could likely serve as a reservoir for *Salmonella* serovars. The study also revealed that *Salmonella* isolates in the poultry environment possess multiple antibiotic resistances due to the presence of antimicrobial genes due to continuous exposure and indiscriminate usage of antibiotics in the area studied. This poses a severe public health threat as it makes therapy difficult, especially in outbreaks. The ability of *Salmonella* to form biofilm and adhere to water systems also poses a severe problem in poultry and humans nearby. Although *Salmonella* in poultry farms cannot be eliminated, it can be controlled if the potential risk factors are managed effectively. Feed trough, water trough, farm size, and farm hygiene could be factors in poultry farms. Therefore, it is crucial to emphasize proper hygiene measures in poultry farms to monitor foodborne salmonellosis constantly.

## Supporting information

S1 File (DOCX)
