# Supplementary material for: Identification and characterization of MDR virulent Salmonella spp isolated from smallholder poultry production environment in Edo and Delta States, Nigeria
Source: PLoS One. 2023 Feb 3;18(2):e0281329. doi: 10.1371/journal.pone.0281329 (PMC9897568; doi:10.1371/journal.pone.0281329)
Supplement: S1 File — (DOCX) [file pone.0281329.s001.docx]

**PLoS ONE**

Supplementary file

**Identification and characterization of MDR virulent *Salmonella* spp isolated from smallholder poultry production environment in Edo and Delta States, Nigeria**

Isoken H. Igbinosa^1,2¶^, Chukwunonso N. Amolo^2¶^, Abeni Beshiru^2, 3¶^, Olajide Akinnibosun^2¶^, Abraham G. Ogofure^2¶^, Maged El-Ashker^4¶^, Mayada Gwida^5¶^, Anthony I. Okoh^6,7¶^ and Etinosa O. Igbinosa^2.3,7¶^*

^1^ Department of Environmental Management & Toxicology, Faculty of Life Sciences, University of Benin, PMB 1154, Benin City, 300283, Nigeria

^2^Applied Microbial Processes & Environmental Health Research Group, Faculty of Life Sciences, University of Benin, PMB 1154 Benin City, 300283, Nigeria

^3^ Stellenbosch Institute for Advanced Study (STIAS), Wallenberg Research Centre at Stellenbosch University, Stellenbosch 7600, South Africa

^4^ Department of Internal Medicine and Infectious Diseases, Faculty of Veterinary Medicine, Mansoura University, Mansoura 35516, Egypt

^5^ Department of Hygiene and Zoonoses, Faculty of Veterinary Medicine, Mansoura University, Mansoura 35516, Egypt

^6^ Department of Environmental Health Sciences, College of Health Sciences, University of Sharjah, Sharjah, United Arab Emirates.

^7^ SAMRC Microbial Water Quality Monitoring Centre, University of Fort Hare, Alice 5700, Eastern Cape Province, South Africa

*Corresponding author e-mail: eigbinosa@gmail.com

^¶^These authors contributed equally to this work

**Running title**: Pathogenic *Salmonella* serovars from poultry**S1 Table**. Oligonucleotides used as primers for PCR in this study

| **Primer** | **Gene** | **Sequence (5′ – 3′)** | **PCR product**  **size (bp)** | **References** |
| --- | --- | --- | --- | --- |
| *Salmonella* spp. | *ST11*  *ST15* | GCCAACCATTGCTAAATTGGCGCA  GGTAGAAATTCCCAGCGGGTACTGG | 429 | [1] |
| *Salmonella*  Typhimurium | *Fli15*  *Tym* | CGGTGTTGCCCAGGTTGGTAAT  ACTCTTGCTGGCGGTGCGACTT | 559 | [2] |
| *Salmonella*  Enteritidis | *Sef 167*  *Sef 478* | AGGTTCAGGCAGCGGTTACT  GGGACATTTAGCGTTTCTTG | 312 | [2] |
| SPVC | *spvC* | F-ACTCCTTGCACAACCAAATGCGGA  R-TGTCTTCTGCATTTCGCCACC | 467 | [3] |
| INVA | *invA* | F-ACAGTGCTCGTTTACGACCTGAAT  R-AGACGACTGGTACTGATCGATAAT | 244 | [4] |
| SDIA | *sdiA* | F-AATATCGCTTCGTACCAC  R-GTAGGTAAACGAGGAGCAG | 274 | [5] |

**S2 Table.** Farm prevalence of *Salmonella* serovars from poultry farms in Edo and Delta State

| **Isolate code** | ***Salmonella* serovars** | **Farm** | **State** |
| --- | --- | --- | --- |
| SE_1_ | *Salmonella* Enteritidis | Mrs. B farm | Edo State |
| SE_2_ | *Salmonella* Enteritidis | One Woman farm | Delta State |
| SE_3_ | *Salmonella* Enteritidis | One Woman farm | Delta State |
| OSS_4_ | *Salmonella* spp | One Woman farm | Delta State |
| SE_5_ | *Salmonella* Enteritidis | One Woman farm | Delta State |
| OSS_6_ | *Salmonella* spp | One Woman farm | Delta State |
| OSS_7_ | *Salmonella* spp | One Woman farm | Delta State |
| OSS_8_ | *Salmonella* spp | One Woman farm | Delta State |
| OSS_9_ | *Salmonella* spp | One Woman farm | Delta State |
| ST_10_ | *Salmonella* Typhimurium | One Woman farm | Delta State |
| ST_11_ | *Salmonella* Typhimurium | One Woman farm | Delta State |
| OSS_12_ | *Salmonella* spp | One Woman farm | Delta State |
| SE_13_ | *Salmonella* Enteritidis | One Woman farm | Delta State |
| ST_14_ | *Salmonella* Typhimurium | Pecas farm | Edo State |
| SE_15_ | *Salmonella* Enteritidis | Mrs. B farm | Edo State |
| SE_16_ | *Salmonella* Enteritidis | Mrs. B farm | Edo State |
| SE_17_ | *Salmonella* Enteritidis | Mrs. B farm | Edo State |
| SE_18_ | *Salmonella* Enteritidis | Mrs. B farm | Edo State |
| ST_19_ | *Salmonella* Typhimurium | Akporido farm | Delta State |
| SE_20_ | *Salmonella* Enteritidis | Akporido farm | Delta State |
| SE_21_ | *Salmonella* Enteritidis | Oputa farm | Edo State |
| SE_22_ | *Salmonella* Enteritidis | Oputa farm | Edo State |
| OSS_23_ | *Salmonella* spp | Cyril farm | Delta State |
| ST_24_ | *Salmonella* Typhimurium | Mrs. B farm | Edo State |
| SE_25_ | *Salmonella* Enteritidis | Pecas farm | Edo State |
| SE_26_ | *Salmonella* Enteritidis | Mrs. B farm | Edo State |
| ST_27_ | *Salmonella* Typhimurium | Pecas farm | Edo State |
| ST_28_ | *Salmonella* Typhimurium | One Woman farm | Delta State |
| OSS_29_ | *Salmonella* spp | Mrs. B farm | Edo State |
| ST_30_ | *Salmonella* Typhimurium | Oputa farm | Edo State |
| ST_31_ | *Salmonella* Typhimurium | Mrs. B farm | Edo State |
| ST_32_ | *Salmonella* Typhimurium | One Woman farm | Delta State |
| OSS_33_ | *Salmonella* spp | One Woman farm | Delta State |
| OSS_34_ | *Salmonella* spp | Pecas farm | Edo State |
| SE_35_ | *Salmonella* Enteritidis | Pecas farm | Edo State |
| SE_36_ | *Salmonella* Enteritidis | Cyril farm | Delta State |
| SE_37_ | *Salmonella* Enteritidis | Cyril farm | Delta State |
| ST_38_ | *Salmonella* Typhimurium | Cyril farm | Delta State |
| ST_39_ | *Salmonella* Typhimurium | Cyril farm | Delta State |
| OSS_40_ | *Salmonella* spp | Cyril farm | Delta State |
| OSS_41_ | *Salmonella* spp | Cyril farm | Delta State |
| OSS_42_ | *Salmonella* spp | Cyril farm | Delta State |
| ST_43_ | *Salmonella* Typhimurium | Cyril farm | Delta State |

**References**

1. Oliveira CJB, Carvalho LFS, Aparecida SF, Tavechio AT, Menezes CCP, Domingues FJJ. Antimicrobial resistance *Salmonella* serotypes isolated from slaughter-age Pigs and environmental samples. Microb Drug Res. 2002; 8(4):407–411.
2. Soumet C, Ermel G, Rose V, Rose N, Drouin P, Salvat G, et al. Identification by a multiplex PCR-based assay of *Salmonella* Typhimurium and *Salmonella* Enteritidis strains from environmental swabs of poultry houses. Lett Appl Microbiol. 1999; 29:1–6.
3. Huehn S, La-Ragione RM, Anjum M, Saunders M, Woodward MJ, Bunge C, et al. Virulotyping and antimicrobial resistance typing of *Salmonella enterica* serovars relevant to human health in Europe. Foodborne Pathog Dis. 2010; 7:523-535.
4. Chiu C, Ou J. Rapid identification of *Salmonella* serovars in feces by specific detection of virulence genes, *invA* and *spvC*, by an enrichment broth culture-multiplex PCR combination assay. J Clin Microbiol. 1996; 34:2619–2622.
5. Halatsi K, Oikonomou I, Lambiri M, Mandilara G, Vatopoulos A, Kyriacou A. PCR detection of *Salmonella* spp. using primers targeting the quorum sensing gene *sdiA*. FEMS Microbiol Lett. 2006; 259(2):201–207.
